# Patient-reported non-adherence and immunosuppressant trough levels are associated with rejection after renal transplantation

**DOI:** 10.1186/s12882-017-0517-6

**Published:** 2017-03-29

**Authors:** Jennifer Scheel, Sandra Reber, Lisa Stoessel, Elisabeth Waldmann, Sabine Jank, Kai-Uwe Eckardt, Franziska Grundmann, Frank Vitinius, Martina de Zwaan, Anna Bertram, Yesim Erim

**Affiliations:** 1 Department of Psychosomatic Medicine and Psychotherapy, University Hospital Erlangen, Friedrich-Alexander University Erlangen-Nürnberg (FAU), Erlangen, Germany; 2 Department of Medical Informatics, Biometry and Epidemiology, Friedrich-Alexander, University Erlangen-Nürnberg, Erlangen, Germany; 3 Department of Nephrology and Hypertension, Friedrich-Alexander University Erlangen-Nürnberg (FAU), Erlangen, Germany; 4 Department II of Internal Medicine, Nephrology, Rheumatology, Diabetes and General Internal Medicine, University Hospital of Cologne, Cologne, Germany; 5 Department of Psychosomatic Medicine and Psychotherapy, University Hospital of Cologne, Cologne, Germany; 6 Department of Psychosomatic Medicine and Psychotherapy, Hannover Medical School, Hannover, Germany; 7 Department of Nephrology and Hypertension, Hannover Medical School, Hannover, Germany

**Keywords:** Kidney transplantation, Rejection, Patient-reported non-adherence, Immunosuppressant trough level variability, Sub-therapeutic immunosuppressant trough levels

## Abstract

**Background:**

Different measures of non-adherence to immunosuppressant (IS) medication have been found to be associated with rejection episodes after successful transplantation. The aim of the current study was to investigate whether graft rejection after renal transplantation is associated with patient-reported IS medication non-adherence and IS trough level variables (IS trough level variability and percentage of sub-therapeutic IS trough levels).

**Methods:**

Patient-reported non-adherence, IS trough level variability, percentage of sub-therapeutic IS trough levels, and acute biopsy-proven late allograft rejections were assessed in 267 adult renal transplant recipients who were ≥12 months post-transplantation.

**Results:**

The rate of rejection was 13.5%. IS trough level variability, percentage of sub-therapeutic IS trough levels as well as patient-reported non-adherence were all significantly and positively associated with rejection, but not with each other. Logistic regression analyses revealed that only the percentage of sub-therapeutic IS trough levels and age at transplantation remained significantly associated with rejection.

**Conclusions:**

Particularly, the percentage of sub-therapeutic IS trough levels is associated with acute rejections after kidney transplantation whereas IS trough level variability and patient-reported non-adherence seem to be of subordinate importance. Patient-reported non-adherence and IS trough level variables were not correlated; thus, non-adherence should always be measured in a multi-methodological approach. Further research concerning the best combination of non-adherence measures is needed.

## Background

Even after a long and frustrating time on the waiting list followed by a successful transplantation, many patients develop problems with adherence to their immunosuppressant (IS) medication. There are different ways to assess non-adherence, e.g. patient-report, family report, physicians’ report, IS medication blood levels, refill record count, electronic monitoring. Depending on the operationalization, the prevalence of non-adherence varies from 2–67% (see reviews of Denhaerynck et al. [1] and Butler et al. [2]). Furthermore, the different non-adherence measures are found to be only partly interrelated [3–7].

When comparing different methods of assessing IS non-adherence, patient-report usually results in higher non-adherence rates compared to other measures [3, 8]; however, even patient-reported non-adherence rates might still underestimate the true non-adherence rate because non-adherent patients could hesitate to admit their true medication taking behavior [9]. Eventhough there is the risk of social desirability with patient-reported non-adherence assessment (as it is with all self-report variables), patient-reported non-adherence has been shown to be associated with rejection [1, 10–12] and graft loss [1, 2, 6, 11, 13]. However, some studies failed to prove this association [6, 14].

Variability of IS trough levels as well as sub-therapeutic IS trough levels have been associated with late rejections and even graft loss in both pediatric and adult patients undergoing different kinds of solid organ transplantation [1, 10, 15–25]. Hsiau et al. [24] as well as Borra et al. [22] reported an association of IS trough level variables and late rejection only for tacrolimus, but not for mycophenolic acid. On the other hand, Fredericks et al. [26] and Tielen et al. [6] did not find tacrolimus level variability to be associated with rejections.

In Germany, psycho-social support of transplant patients is one of the objectives of psychosomatic care, including also the optimization of medication adherence as a part of health behavior. As measuring non-adherence via patient-report might be distorted by social desirability and electronic medication monitoring systems are expensive and impractical for clinical routine, we intended to investigate if variability of IS trough levels as well as sub-therapeutic IS trough levels might be easy assessable objective parameters of patients’ medication adherence behavior and also a potential outcome measure for adherence trainings.

The aim of the present study was to investigate whether graft rejection after renal transplantation is associated with patient-reported IS medication nonadherence and/or IS trough level variables (IS trough level variability and the percentage of sub-therapeutic IS trough levels) and whether a combination of both would result in the best prediction.

## Methods

### Patients

This multi-center study was performed at the transplant centers of the University Hospitals of Erlangen and Cologne and of Hannover Medical School and included 267 consecutive adult patients who had undergone renal transplantation between 1981 and 2014. Inclusion criteria encompassed:

1. being ≥12 months post transplantation,
2. availability of ≥ 4 IS trough levels within the last 12 months before the non-adherence assessment; levels within the first 6 months after transplantation were not included; thus, in patients who were 12 months after transplantation, through levels of only 6 months were included,
3. no additional non-renal transplantation,
4. no severe mental disorder,
5. no cognitive impairment, and
6. sufficient German language skills for understanding of the questionnaires.

Institutional ethic board approval was obtained from the Clinical Ethics Committee of the University Hospital Erlangen (Friedrich-Alexander-Universität Erlangen-Nürnberg (FAU)), the Clinical Ethics Committee of Hannover Medical School, and the Ethics Committee of the University Hospital of Cologne. All participants gave written informed consent.

Assessment of patient-reported non-adherence was conducted between November 2014 and December 2015 when participants were at the respective transplant centers for routine follow-up examinations. Trained medical staff, who were not part of the transplant team, approached the patients and distributed the questionnaires. IS trough levels and biopsy-proven graft rejections from the last maximal 12 months before the non-adherence assessment were recorded from the patient charts.

### Assessment of patient-reported IS medication non-adherence

For the assessment of patient-reported IS medication non-adherence, the Basel Assessment of Adherence to Immunosuppressive Medications Scale (BAASIS©; [27]) was used. It consists of four items concerning adherence to IS medication (dose taking, drug holidays, timing deviation >2 h from prescribed time, dose reduction) to be rated on a 6-point scale (0 = never, 5 = every day). Non-adherence is defined as at least one affirmative answer to any of the four items (dichotomous score). Following a systematic review of self-report instruments to identify medication non-adherence, the BAASIS© was recommended as a reliable, valid, and sensitive tool [27].

### IS trough levels

We assessed two variables concerning IS medication (tacrolimus, cyclosporine, everolimus, sirolimus) trough levels: IS trough level variability and percentage of sub-therapeutic IS trough levels. IS trough level data were assessed from ethylenediamine tetraacetic acid (EDTA) blood samples via LC-MS/MS (liquid chromatography mass spectrometry/mass spectrometry) in Erlangen and Hannover and via immune-assays in Cologne. IS trough levels that were measured during hospitalization or apparently shortly after IS intake were excluded as invalid. The minimal number of valid IS trough levels for calculation of IS trough level variability and percentage of sub-therapeutic IS trough levels was four, because the medication levels from several time points are thought to be more valid than a single medication level from one time point [10].

IS trough level data were standardized for the individual target level by dividing every single trough level by its respective target level. The target level is determined individually for each patient and can be changed by the transplant physicians depending on the clinical course. Thus, the target levels can differ both inter-patient and intra-patient. By dividing every single level by the respective target level, we controlled for changing target levels and excluded variation due to those changes. For these standardized trough levels, means (M) and standard deviations (SD) were calculated. We used intra-patient coefficients of variation (CV) as a measure for *IS trough level variability*, as suggested by Hsiau et al. [24]. The CV was calculated by dividing the SD by the respective M. The higher the CV, the more erratic are the IS trough levels. The *percentage of sub-therapeutic IS trough levels* was calculated by dichotomizing every single IS trough level for each patient (< or ≥ the respective target level) and determining the proportion of sub-therapeutic IS trough levels for each patient (0–100%).

### Late rejections

All patients were transplanted ≥12 months prior. Late rejection was defined as any biopsy-proven acute rejection ≥6 months after transplantation. Rejections that occurred more than 12 months before the patient-reported non-adherence assessment were excluded from the analysis. Thereby, we analyzed an assessment period of 12 months, which began 6 months to 34 years after the last transplantation.

### Statistical analyses

In the case of missing variables the patient was excluded from the respective analysis. Two patients (0.7% of the whole sample) did not provide self-report non-adherence data. For an overview, M, SD, ranges, and frequencies (as appropriate) for all variables are given. A logistic regression model was conducted with rejection being the dependent variable and patient-reported non-adherence, IS trough level variability, percentage of sub-therapeutic IS trough levels, and age at transplantation as independent variables. Preliminary analyses comprised Pearson, point-biserial and Spearman correlation coefficients as well as group comparisons (Mann-Whitney-U-tests and Chi^2^-tests). Statistical analyses were conducted with the statistical analysis program SPSS 21. Findings were considered to be statistically significant at *p < 0.05*. Due to the explorative character of the study, no correction for multiple testing was conducted.

## Results

### Socio-demographic and biomedical data

A total of 267 patients were included in the analyses (35% females); the mean age at assessment was 52.8 years and the incidence of late allograft rejection 13.5%. For detailed information, please see Table 1.

**Table 1:** Socio-demographic and biomedical data

		All patients (*N* = 267)
Sex (females: *n*, %)		93 (35%)
Age (in years; M, SD, range)		52.8 ± 13.7 (18–80)
Age at transplantation (in years; M, SD, range)		45.8 ± 14.7 (2–76)
Time since last transplantation (in years; M, SD, range)		7.1 ± 6.2 (1–34)
Dialysis before transplantation (yes: *n*, %)		238 (89.1%)
Immunosuppressive medication (*n*, %)	Tacrolimus	180 (67.4%)
	Cyclosporine	59 (22.1%)
	Sirolimus	22 (8.2%)
	Everolimus	6 (2.2%)
Number of renal transplantations (*n*, %)	1	233 (87.3%)
	2	26 (9.7%)
	3	8 (3.0%)
Number of IS trough levels (M, SD, range)		9.6 ± 4.3 (4–37)
IS trough level variability (M, SD, range)		21.3 ± 10.2 (2.7–56.1)
Percentage of sub-therapeutic IS trough levels (M, SD, range)		42.3 ± 27.6 (0–100%)
Patient-reported non-adherence (*n*, %)		89 (33%)

### Correlation analyses and group differences

IS trough level variability, percentage of sub-therapeutic IS trough levels, and patient-reported non-adherence were all three significantly correlated with late allograft rejection.

Patients with and without rejections did not differ significantly from each other regarding sex, time since last transplantation, dialysis before transplantation, IS medication type and number of IS trough levels, but did differ regarding age and age at transplantation. Therefore, age and age at transplantation were also considered as potential independent variables for regression analyses.

To avoid problems of multicollinearity in the following regression analyses, we conducted correlation analyses between the independent variables: IS trough level variability, percentage of sub-therapeutic IS trough levels, and patient-reported non-adherence were not correlated with each other. Patient-reported non-adherence (BAASIS) was significantly correlated with age and age at transplantation. However, r was <0.150, so we assumed the correlation to be small enough to be negligible for the regression analysis. As was to be expected, age and age at transplantation were significantly correlated with each other (*r* = 0.908, *p < 0.001*). Consequently, we decided to include only one variable in the regression analysis. We chose age at transplantation, because its correlation with rejection was somewhat stronger (−0.246, *p < 0.001* for age at transplantation versus −0.197, *p = 0.001* for age).

### Regression model

The full model including all independent variables explained 18.9% of the variance. Age at transplantation and the percentage of sub-therapeutic IS trough levels reached significance, whereas IS trough level variability and patient-reported non-adherence did not. The odds ratio was particularly high for the percentage of sub-therapeutic IS trough levels (OR 6.136; 95% CI 1.524–24.708). Including patient-reported non-adherence increased the amount of explained variance only non-significantly by 1.4%. For details, please see Table 2. The results did not change, when the regression analysis was repeated by including only tacrolimus medication. For the other IS medication groups, the samples were too small to conduct separate regression analyses.

**Table 2:** Logistic regression model for the prediction of rejection including age as predictor

	Regression coefficient B (standard error)	Wald	Odds ratio	95% confidence interval	*p* single predictors	Explained variance (Nagelkerke’s R^2^) whole model	*p* whole model	Delta *p*	−2 Log-Likelihood
Age at transplantation	−0.047 (0.013)	12.581	0.954	0.929–0.979	0.000	0.112	<0.001		193.810
Percentage of sub-therapeutic IS trough levels	1.814 (0.711)	6.515	6.136	1.524–24.708	0.011	0.160	<0.001	0.006	186.339
IS trough level variability	0.029 (0.018)	2.566	1.029	0.994–1.066	0.109	0.175	<0.001	n.s.	183.941
Patient-reported non-adherence	0.586 (0.387)	2.292	1.796	0.841–3.835	0.130	0.189	<0.001	n.s.	181.679

## Discussion

Our main findings are as follows: (1) IS trough level variability, percentage of sub-therapeutic IS trough levels, and patient-reported non-adherence were all three associated with rejection, but not with each other. (2) Logistic regression analysis revealed that rejection was significantly associated with a higher percentage of sub-therapeutic IS trough levels and younger age at transplantation.

The rate of late allograft rejection within the last 12 months (6 months to 34 years after the last transplantation) was 13.5% in our sample and therefore comparable to other studies. Three studies with assessment periods of 12–24 months (11 months to 15 years post transplantation; excluding the first 6 months post transplantation) reported rejection rates of 9.9–10.1% [15, 17, 18]. Rejection rates of 10.5–11.2% were reported by two studies with assessment periods of 12 months (1–7.7 years post transplantation; under inclusion of the first 6 months post transplantation) [22, 26]. In contrast, rejection rates from 21.7 to 42.4% were found in some studies [6, 16, 20, 21, 23–25], but these might be due to longer assessment periods than in our study and the inclusion of the first 6 months after transplantation (0.5–8.5 years after transplantation), when most complications and especially most acute rejections occur.

When comparing IS trough level variability in different studies, it has to be kept in mind that different measures have been used for the calculation of IS trough level variability. Several researchers used the SD to express IS trough level variability [3, 16–18, 20, 23, 24, 26]. The problem with SD is that it skews toward individuals with higher mean IS trough levels [24]. Therefore, a standardization with the mean, i.e. the CV, is preferable [15, 19, 21, 24, 25, 28] and has also been found to be more reliable [24]. By using standardization by the target level instead of the unstandardized blood levels we excluded variation due to changing IS target levels for medical reasons [10] in the present study. Other studies have also corrected for drug dosage, but the formula was not specified in detail [21, 22]. These different approaches to measure IS trough level variability must be taken into account when comparing different studies. We found a mean IS trough level variability (CV) of 21.26%, with a SD of 10.16% ranging from 2.74 to 56.08% (median 19.67%). Compared to other studies using the CV, this was quite low [15, 21, 24, 25, 28]. Similarly to our results, Tielen et al. [6] found that IS trough level variability was not associated with patient-reported non-adherence (BAASIS).

Apart from IS trough level variability, the percentage of sub-therapeutic IS trough levels was particularly associated with late allograft rejection. Also other investigators have found low/sub-therapeutic IS trough levels to be associated with late rejection [20, 25] and even graft loss [15].

That self-reported non-adherence is only correlatively associated with rejections, but no longer in regression analyses (outperformed by age and the percentage of sub-therapeutic IS trough levels) might be due to non-adherence self-reports being contaminated by memory biases and social desirability and to rejections being not only influenced by non-adherence but also by variables like sub-therapeutic IS trough levels. Similarly, the lack of association between patient-reported non-adherence and IS trough level variables is likely due to the fact that the IS trough level variables do not only display non-adherence behavior, but can also be caused by drug-drug interactions or other pharmacokinetic factors [17, 18, 29–33]. Patient-reported non-adherence is not equivalent to a high IS trough level variability or to a high percentage of sub-therapeutic IS trough levels, but is an important reason for a high variability and/or sub-therapeutic levels, that needs to be recognized. Therefore, also the percentage of sub-therapeutic IS trough levels might be a potential outcome measure for adherence trainings though it is not associated with patient-reported non-adherence.

When comparing the rate of patient-reported non-adherence (BAASIS) in our study, similar results were obtained in other studies in adult [8, 10] and pediatric [11] renal transplant recipients (30–40%). Lennerling et al. [34] even found 54% of the patients to be non-adherent according to the BAASIS. Yet, it has to be kept in mind that these data might be subject to a recruitment bias (non-adherent patients might be more likely to refuse study participation than adherent patients) and/or social desirability.

Younger age is considered to be a risk factor for poor adherence when comparing adolescents with adults [1, 20], this might be due to adolescence-related issues like concerns about body image, developmental tasks related to autonomy and self-identity, cognitive development (abstract thinking, behavioral control and responsibility), and risk taking / experimentation [35, 36]. Our study was conducted only in adults and revealed that patients, who experienced rejection, received renal transplantation at a younger age than patients with no rejection (36.6 ± 15.7 years vs. 47.2 ± 14.0 years old at the time of transplantation). Similarly, Borra et al. [22] reported recipient age at transplantation to be a significant risk factor for poor long-term outcome in adult renal transplant recipients (mean recipient age at transplantation was 41 years for the kidney failure group and 48 years for the control group). Younger adult age at transplantation might be associated with rejection and also with poorer patient-reported adherence due to a lower perceived threat of disease and multiple stressors regarding family and professional activity in younger years. Yet, our data are not sufficient to finally show whether non-adherence contributes to the poorer outcome of younger patients.

There are several limitations to our study. Firstly, our study is retrospective (IS trough level variability, sub-therapeutic IS trough levels) and cross-sectional (patient-reported non-adherence). Thus causal interpretations should be made with caution. Furthermore, IS trough level variability and sub-therapeutic IS trough levels were assessed both before and after rejection and rejections can be followed by IS dose adjustments and might change IS trough level variables and also patient-reported non-adherence. Yet, we would expect this fact to *minimize* associations between rejections, patient-reported non-adherence, IS trough level variability, and sub-therapeutic IS trough levels. Therefore, the association could even be stronger in prospective studies (beginning with the assessment of self-reported non-adherence). Secondly, we did not assess other risk factors of acute rejection, such as the presence or new onset of infections by which drug exposure could be influenced. Thirdly, we only considered the IS medications to be regularly measured in the three transplant centers and ignored additional IS medications (e.g. mycophenolate mofetil, cortisone) and non-IS medications (e.g. antihypertensive drugs). Furthermore, IS trough level data were assessed with different methods (via LC-MS/MS in Erlangen and Hannover and via immune-assays in Cologne). However, these different methods should have negligible effects because of the standardization by target levels and calculation of the CV. Finally, we did not use electronic medication monitoring systems as a more objective reference method for non-adherence. Such methods are considered as closest to a gold standard in measuring non-adherence, but they are expensive and impractical for clinical routine [10].

## Conclusion

Sub-therapeutic IS trough levels, high IS trough level variability and patient-reported non-adherence are each associated with late acute rejection in renal transplant patients. However, they are not significantly associated with each other. Particularly, the percentage of sub-therapeutic IS trough levels seems to be strongly associated with acute rejections after kidney transplantation whereas IS trough level variability and patient-reported non-adherence seem to be of subordinate importance. Patient-reported non-adherence and IS trough level variables seem to measure different facets of non-adherence and cannot replace each other. Consequently, non-adherence should always be measured in a multi-methodological approach. Further research concerning the best combination of non-adherence measures is needed. Moreover, younger age at transplantation should be considered as a risk factor of rejection.

## Abbreviations

BAASIS: Basel Assessment of Adherence to Immunosuppressive Medications Scale
CV: Coefficient of variation
EDTA: Ethylenediamine tetraacetic acid
IS: Immunosuppressant
M: Mean
SD: Standard deviation
